# Algorithmic Approach to Inflammatory Disorders of Ileum

**DOI:** 10.30699/IJP.2022.539357.2736

**Published:** 2022-09-10

**Authors:** Afshin Moradi, Ali Mokhtarpour, Amir Yazdani, Kianoosh Kianersi, Leila Bahari Khasraghi

**Affiliations:** *Men’s Health and Reproductive Health Research Center, Shahid Beheshti University of Medical Sciences, Tehran, Iran*

**Keywords:** Acute ileitis, Algorithmic approach, Chronic ileitis, Crypt architecture, Histological patterns, Ileal biopsy interpretation

## Abstract

The ileum has been candidate more frequently for endoscopic biopsy compared to the past. Most of those biopsies show either completely normal tissue or non-specific changes. Nevertheless, in some diseases, ileal biopsy would be diagnostic, and in some cases, it may be the only anatomical involved location by the disease. Endoscopically, normal mucosal biopsy is unlikely to provide useful diagnostic information and is not routinely recommended. However, in the presence of ileitis, ulcers, or erosions, biopsies can be very helpful. Ileitis might be induced by various conditions including infectious diseases, vasculitis, medication-induced, ischemia, eosinophilic enteritis, tumors etc. The conclusive cause of the condition is proposed by a comprehensive clinical background and physical examination, laboratory investigations, ileocolonoscopy, and imaging findings. Ileoscopy and biopsy are mainly useful in correctly selected cases such as patients who present with inflammatory diarrhea and endoscopic lesions. The purpose of this review article is to provide a simple algorithmic approach to the ileal biopsy samples through several boxes that give diagnostic clues and an idea behind the categories of ileal disorders.

This review is written based on those that were previously reported in the literature as well as the authors' experiences. We have summarized different histological patterns in the ileal biopsy specimens that can be used in the diagnosis of inflammatory disorders of the ileum.

This review provides an algorithmic approach to the clinicopathological features of inflammatory disorders of the ileum with a brief discussion of some important related issues.

## Introduction

Terminal ileum endoscopy and sampling are acknowledged as the most reliable methods to detect chronic enteritis. The observations of endoscopic manifestation of the disease and/or a clinical manifestation of inflammatory diarrhea are the main implications for the sampling. Pathologists often deal with small intestinal samples regularly and are required to be familiar with the various morphological patterns and the potential differential diagnoses of the diseases. The small intestine is not a usual site for the gastrointestinal tract malignancies, especially carci-nomas. However, it is a relatively frequent site of primary gastrointestinal lymphoma, and results of a recently published study showed that the small intestine is the second most prevalent location for the primary lymphoma, after the stomach (1).

The most important implications of the terminal ileum sampling include presence of the lesions in the setting of “inflammatory diarrhea” and Crohn's disease. Terminal ileum endoscopy and biopsy are considered as the main diagnostic tools to diagnose the conditions. A regular sampling of the terminal ileum is barely yielding information in the patients presenting with chronic non-bloody diarrhea and unremarkable ileoscopy. Besides, it may give useful data in specific cohort of the patients since it is able to detect primary villous atrophy of the ileum and epithelial lymphocytosis of the ileum in individuals with the aberrant laboratory findings. Essentially, in an unselected group of individuals, the diagnostic accuracy of the ileal endoscopy is about 5% and the diagnostic microscopic finding is found in only 0–0.6% of individuals with the chronic diarrhea and normal ileoscopy (2).

The definite number of biopsies that have to be taken in the terminal ileum has not yet been determined. Nonetheless, it has been suggested to collect multiple tissue samples (at least two samples) (3, 4). 

 Pathologists need to know about the heterogeneity of the biopsies, even when taken from the unremarkable ileum, due to the existence of the Peyer’s patches. These lymphoid areas are centralized in the distal 25 cm of the ileum but can reach out up to 200 cm proximally. They are principally located in the terminal 10–15 cm where they set up a lymphoid ring. The Peyer's patches are more prominent in children and adolescents and tend to diminish as the individual gets older (5).

**Anatomy and Histology**

The ileum has numerous characteristic features, comprising its particular junction with the colon, a large content of the lymphoid tissue, and deposits of pigment. 

The mucosal features of the ileum, compared to both jejunum and duodenum, encompass shorter and less number of the plicae circulars and a larger population of the goblet cells. The villi are shorter and display fewer serrations than they do at more proximal sites, and usually show a finger-like morphology. They are much less linear structurally, which make tangential cuts appear even in normal samples. These morphologic patterns become steadily more obvious throughout the length of the small bowel and are most obvious in the distal ileum.

**Ileal Mucosal Architecture**

The mucosal epithelium covers the villi and crypt structures. Each villous is surfaced by one layer of epithelium of various kinds: the columnar epithelial cells and the goblet cells. The columnar cells are regularly present in the proximal part of the small bowel. Differently, the goblet cells are repeatedly placed in the distal part.

The endocrine cells are found throughout the villous epithelium, but their population is increased within the crypts. The crypt epithelium is principally responsible for the epithelial cell restoration (6).

Intraepithelial lymphocytes (IELs) are located among the epithelial cells on the top of the basement membrane. The crypts contain IELs, but neutrophils and plasma cells are not generally seen. 

The discriminating mucosal features of the terminal ileum comprise an increased number of the goblet cells, rather shorter villi in comparison with the jejunal villi, and the existence of differentiated groups of the Peyer's patches found mostly in the mucosa.

In children, they can be seen macroscopically adjacent to the ileocecal junction (7). 

Architecturally, four discrete elements could be seen in the Peyer's patches: the follicle, the dome, the interfollicular area, and the follicle-related epithelium (8). 

Lymphoid follicles wrap a germinal center occupied by the IgA-positive B cells with infrequent CD4-positive T cells and macrophages enveloped by a mantle zone, which is populated by the small IgD- and IgM-positive B cells. The dome is the region between the follicle and the overlying epithelium. It contains macrophages, B cells and plasma cells.

The follicle-associated epithelium, which covers lymphoid cells has a smaller number of the goblet cells and displays low cuboidal and flattened epithelium, commonly named as M cells. The M cells promote the interaction between the antigens present in the lumen and lymphoid cells within the mucosa. The notable lymphoid population between the follicles is the fourth part of the Peyer's patches and is associated with the T cell-rich interfollicular area.

The brown-black granular pigment is ordinarily found deep down the Peyer's patches or in the lamina propria of the ileum in adults within the macrophages. The pigment is not important and is probably derived from the aerial particles or food (9).

The mucosa from the ileum to the colon displays a fall of villi seen at various lengths across the intracecal segment of the ileum. 

The cells of the lamina propria consist of lymphocytes, eosinophils, monocytes/macrophages, mast cells, connective tissue cells, vessels, and nerve endings as well as smooth muscle cells of the muscularis mucosae.

Above the lymphoid aggregate, IELs are typically abundant. The majority of them are T lymphocytes with a suppressor phenotype. The lamina propria lymphocytes consist of B and T cells and a smaller number of natural killer cells (NKCs). Plasma cells constitute the principal subtype of B cells. Most of the T cells are CD4+ helper cells.

The histiocytes population is less than lymphocytes or plasma cells. They are regularly present in the upper portion of the lamina propria beneath the small superficial blood vessels underneath the subepithelial collagen (10).

On the basis of these aggregates, the intestinal macrophages can be grouped into the pigmented and nonpigmented macrophages. The pigmented ones encompass *Pseudomelanosis coli*, air-borne dust, barium deposits, and hemosiderosis; the non-pigmented or foamy kinds pose a differential diagnostic dilemma of muciphages, lysosomal storage diseases, and infections such as Whipple's disease and *Mycobacterium avium *complex infection. Muciphages are mucin-rich macrophages which are seen in cases with mucosal damage, frequently present in the last step of repair following preceding injury and/or minor insult (11).

Neutrophils are not normally seen out of the capillary lumen. More than three extravasated neutrophils within the lamina propria, is regarded as a pathologic finding (12).

To see the abnormal histological features, being familiar with the normal ileal histology is required (Table 1).

**Table 1:** Key Points to remember in normal ileum histology

**Distinctive features of ileal mucosa are the presence of more goblet cells on the surface, short villi, and the Peyer’s patches.**
**Granular brown-black pigment is common in the deep portions of Peyer’s patches or in the lamina propria of the ileum, notably in adults. The pigment can accumulate within macrophages.**
**IELs count of more than 40 is considered abnormal.**
**The presence of more than three extravasated neutrophils in the lamina propria would be regarded as a pathologic finding**.

**Figure:**
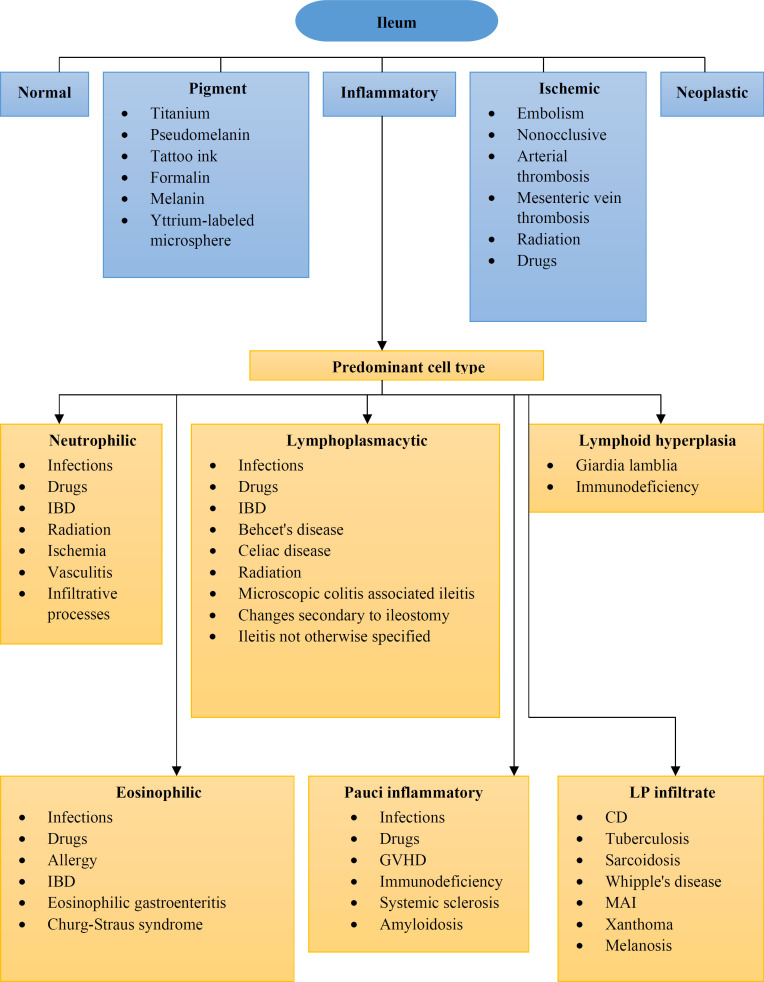


**Clinicopathological Correlation**

In general, there are three recognized groups of the clinical situations related to the inflammatory lesions of the ileum:

1. Ileitis with no or minor gross abnormalities

Pathologic changes of the terminal ileum, comprising shortening of the villus, flattening of the superficial epithelial cells, and increased IELs, are present in nearly 15% of the individuals with lymphocytic and collagenous colitis in comparison with the control groups. A small number of the cases related to the microscopic colitis with ileal villous atrophy were evaluated as the primary lesions.

1. Isolated ileitis

The patients with tiny ulcers in the terminal ileum without lesions in the colon or ileocecal valve (isolated terminal ileal ulcers) may present clinical symptoms or stay symptom-free. The pathogenesis of these lesions, as well as the exact nature of the microscopic “focal” or “isolated active ileitis”, pose a diagnostic dilemma. Drugs, infections, and idiopathic inflammatory bowel diseases need to be taken into consideration.

1. **Ileocolitis**

**Acute-Active Disease**

Acute enteritis and ileitis are usually infectious in their nature. Most of the intestinal infections (90%) lead to the non-specific mucosal injures. The majority of the infections are self-limited and histopathologic investigation is not necessary for the diagnosis.

**Chronic Ileitis**

**Infections**

Many kinds of the infections have a chronic course. The granulomas are mainly seen in *Yersinia Pseudotuberculosis* infection. The intestinal tuberculosis is mainly induced by *Mycobacterium tuberculosis* and less commonly by *Mycobacterium bovis*, the later rarely occurs in the Western world due to the pasteurization of milk. Both microorganisms prefer the small intestinal involvement, in particular, the terminal ileum.

**Drugs**

Different kinds of drugs can develop symptoms associated with pathologies in the small intestine. NSAID-related small intestinal lesion is a well-known drug-induced inflammatory pathology of the intestine.

**Crohn's Disease (CD)**

The presence of lesions in the ileum is the hallmark of the condition that provides a distinction between CD and UC. The microscopic lesions could be very different. They might be trivial and focal in the background of unremarkable mucosa or expanded. Overall, necrosis of the surface epithelium is quite frequent in the early stage of the lesions in Crohn's disease, while cryptic epithelial cells are not commonly affected. The significant useful microscopic findings include structural disturbances of the villi (irregularity and blunting or widening), retained mucin secretion or over-production of mucin by the epithelial cells, pseudopyloric metaplasia, uneven spread of the Paneth cells within the crypts, active chronic inflammation, and the formation of granulomas.

**Behçet's Disease**

Behçet's disease is commonly presented with a single or a number of punched-out ulcers that are often large, round to oval with a well-delineated edge and rather a flat base. The lesions are mainly situated on the opposite side of the mesentery.

**Terminal Ileitis and Ulcerative Colitis (UC)**

Inflammation is noted in the terminal ileum in less than 17% of individuals affected by UC. The inflamed areas are frequently limited to the terminal 10 to 15 cm of the ileum (″backwash ileitis″).

It could also be an early presentation of the ulcerative colitis, which may also involve the upper part of the gastrointestinal tract (13-15).

For an algorithmic approach to the ileal biopsy, we should categorize the histological findings into several major patterns (Table 2).

**Table 2:** Major categories of nonneoplastic disorders of ileum patterns

**Acute (active) ileitis pattern**
**Chronic ileitis**
**Lymphoid hyperplasia pattern**
**Eosinophilic pattern**
**Ischemic ileitis pattern**
**Pauci inflammatory pattern**
**Lamina propria infiltrate**
**Pigments and extras**

**Acute (Active) Ileitis (Enteritis) Pattern**

Acute inflammation in the ileum has somehow similar features to the other parts of the gastrointestinal tract (Table 3).

**Fig. 1:**
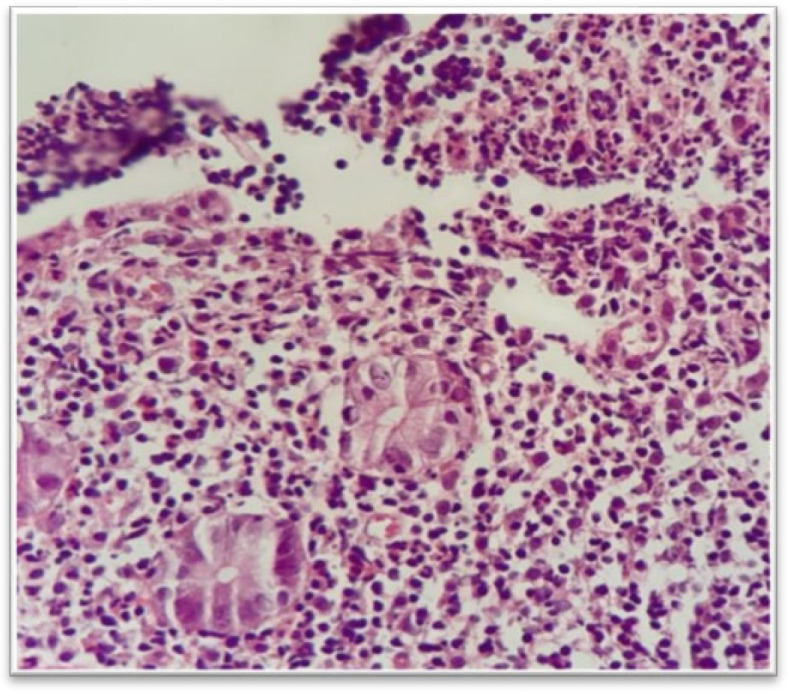
Aphthous ulcer, H&E stain, lymphoid follicles with surface erosion (upper right) (40 x).

Aphthous ulcer is defined as a lymphoid follicle with the surface erosion (Figure 1).

The most common etiology is infection. Involvement of the ileum may be an isolated process or commonly affects the stomach and upper part of the small bowel, including the duodenum and jejunum, or the colon.

Histology is usually neither necessary and nor helpful for the diagnosis of the acute infectious ileitis (16, 17).

The etiological associations of the acute ileitis are outlined in Table 4.

**Chronic Ileitis (Active /Inactive)**

The chronic ileitis is defined by the multiple morphological features (Table 5).

**Fig. 2:**
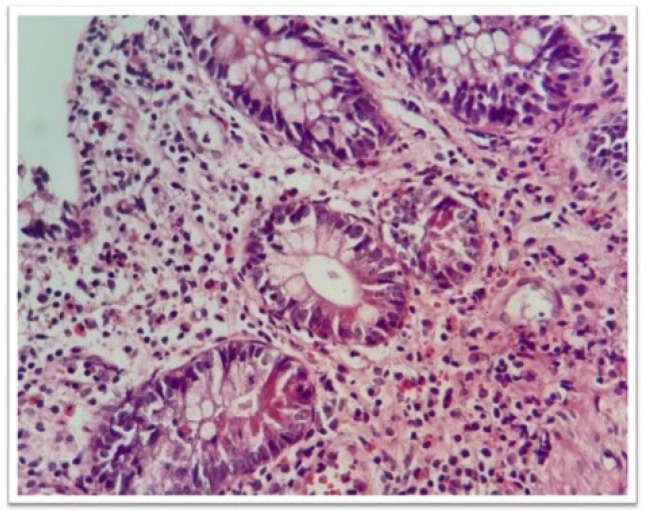
Chronic ileitis with mucosal fibrosis, H&E staining. Deep mucosal fibrosis (lower right) above the muscularis mucosa (40 x)

Fibrosis and granulomas are important features of the chronic inflammation (Figure 2).

Interpretation of the ileal biopsies needs a systemic approach. First of all, we have to specify whether (or not) the chronic ileitis is accompanied by the activity. Based on this, the relevant differential diagnosis should be considered (Table 6).

**Table 3:** Terms, definitions, and features of activity in ileum

Epithelial cell changes **Epithelial degeneration** **Epithelial regeneration**
Erosion/ulcer
Cryptitis **Normally, each crypt should not show >1 polymorphonuclear cell infiltrate, and infiltrates more than this, indicating the activity.**
Crypt abscess
Loss of goblet cells ** Number of goblet cells compared to columnar enterocytes ranging from 32 to 50 goblet cells per 100 epithelial cells.**
Apoptosis
Inflammatory cells composition and distribution **Neutrophils** **Eosinophils (intraepithelial)**
Lamina propria changes ** Edema** ** Hemorrhage** ** Necrosis **

**Table 4.** Acute (active) ileitis pattern: Etiologic Associations

Medications
Infection
Bacterial
** Salmonella** ** Shigella** ** Enterotoxigenic ** ***Escherichia coli*** ** Yersinia enterocolitica** **Yersinia paratuberculosis**
Viral
**CMV**
Fungal
**Candidiasis** ** Mucormycosis**
Parasitic
**Amoebic**
Idiopathic Inflammatory Bowel Disease
Infiltrative Processes
Radiation injury
Ischemia
Vasculitis

**Table 5:** Terms, definitions, and features of chronicity in ileum

Architectural changes
Villi: **Variability in size and shape, Shortening, broadening of villous tips, Irregularity, Loss** Crypt distortio**n: Branching, Shortening, atrophy, loss, Irregular distance between the crypts**
Epithelial cell change
Metaplasia: **Pyloric, gastric surface metaplasia**
Goblet cell rich crypts (hypercrinia or goblet cell hyperplasia)
**An increase beyond 50% of the normal range (up to 50 goblet cells per 100 epithelial cells), occurs in Crohn’s disease but can also occur in other conditions**
Paneth cells loss or hyperplasia
Inflammatory cells composition and distribution
**Basal plasmacytosis ** **Mucosal (basal) and submucosal lymphoid aggregates**
**Granulomas**
**Fibrosis**

**Table 6:** Chronic ileitis pattern: Etiologic Associations

Idiopathic Inflammatory Bowel Disease
**Crohn’s Disease & Ulcerative colitis**
Infections
**Persistent infections** **Tuberculosis** **Yersinia**
Drugs
**NSAIDs** **Chemotherapeutic agents** **Sprue-like or AIE-like mucosal inflammatory process (Ipilimumab, PD-1/PD-L1 inhibitors, Olmesartan)**
Radiation-induced changes
Crohn’s disease-like
Microscopic colitis associated ileitis
Behçet’s Disease
Non-specific isolated ulcers of the terminal ileum
Ileitis not otherwise specified
Changes secondary to the ileostomy
Underlying mass lesions ** Primary tumors, metastatic malignancies, endometriosis**

**Infection**

Several infectious diseases have chronic courses, such as yersiniosis, tuberculosis and typhoid fever.

Yersiniosis shows a combination of inflammatory cells including neutrophils, plasma cells and histiocytes with a granulomatous reaction. It has four stages:

1- Lymphoid hyperplasia with germinal centers

2- Histiocytic infiltration

3- Large geographic epithelioid granulomas 

4- Central necrosis of granuloma

*Mycobacterium tuberculosis*, followed by *Mycobacterium bovis* are the most prevalent etiologies of the intestinal tuberculosis. The terminal ileum is the major site of involvement in the GI tract. Tuberculosis is characterized by the multiple large confluent caseating granulomas with the cuffs of lymphocytes, ulcers covered by epithelioid histiocytes, submucosal inflammation without edema, and fissures. In the chronic stages, fibrosis and hyalinized vaguely granulomas are predominant. Round ulcers and firm sessile polyps were seen in the endoscopic study. Culture of the biopsies is needed to diagnose the intestinal tuberculosis.

The specific histological patterns could be associated with the bacterial infections (Table 7).

Typhoid fever leads to the lymphoid hyperplasia of the Peyer's patches and mesenteric lymphadenitis. Large macrophages digesting intracellular organisms called typhoid cells or Mallory cells are noted in the lymphoid aggregations. Neutrophils are rare. Tissue necrosis is present even in the muscularis propria and vascular thrombosis. The whole length of the ileum and colon might be inflamed and congested. Longitudinal ulceration is seen especially in the ileum (18-21).

**Drugs**

Several drugs can cause symptoms and pathological changes in the small intestine. The well-known drug-induced inflammatory enteropathy is created by NSAIDs. Chemotherapeutic agents can induce epithelial cell apoptosis and villous atrophy. Ipilimumab, Idelalisib, and Pembrolizumab may lead to a sprue-like or autoimmune enteropathy-like small bowel inflammatory disease. Olmesartan gives rise to the villous blunting, increased apoptosis of the crypts, intraepithelial neutrophils and lymphocytes, and increased lamina propria cells. The chronic pathologic changes are less common in the NSAID-injuries (22-24).

**Crohn's Disease**

**Fig. 3:**
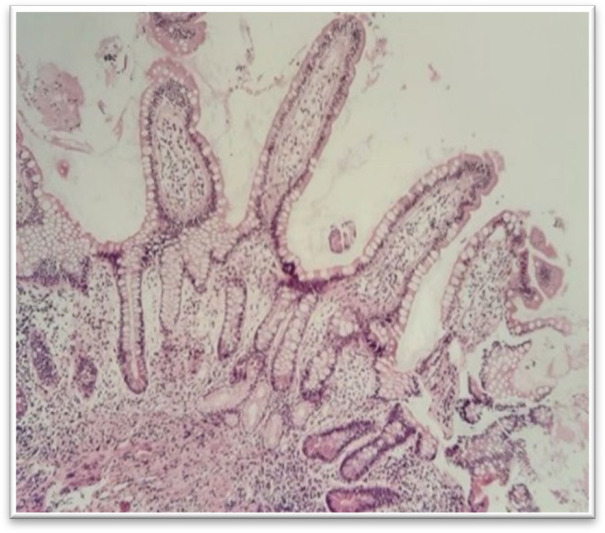
Pyloric gland metaplasia, H&E staining. Pyloric gland metaplasia is a sign of chronic inflammation related to mucosal damage. It is not a specific marker (10 x)

**Fig. 4:**
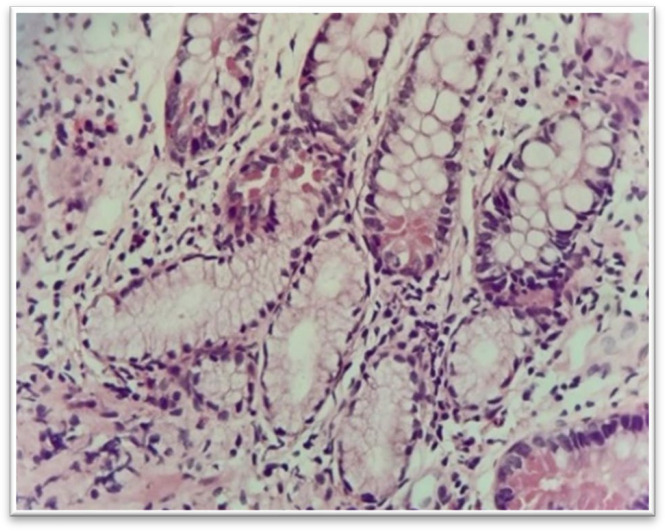
Pyloric gland metaplasia, H&E stain (40 x)

The ileal involvement is an important finding to differentiate between CD and UC. The microscopic features are variable based on the disease severity and the site of sampling. They might be minimal and focal in a background of unremarkable mucosa, or they may be widespread. The main diagnostic microscopic findings are crypt and villous abnormalities, including irregularity and blunting or broadening of the villi, maintained or increased mucin secretion by the epithelial cells, pseudopyloric metaplasia, irregular distribution of the Paneth cells in the crypt, chronic active inflammation in the lamina propria with neutrophilic cryptitis, crypt abscess, surface erosion and ulceration, and the presence of fibrosis and granuloma (Figures 3 and 4).

These changes are usually seen in a patchy or segmental form (25, 26).

**Table 7:** Specific histologic patterns of bacterial infections

Lympho-histiocytic pattern
**Salmonella. typhimurium**
Pseudomembranous pattern
**Clostridium difficile**
Diffuse histiocytic pattern
**Rhodococcus equi** **Mycobacterium avium in immune-compromised patients** **Histoplasmosis**
Granulomatous pattern
**Tuberculosis** **Yersinia infection**

Ulcerative Colitis

Grossly the mucosa seems diffusely reddish and granular with prominent erosion. In the mild form of the disease these findings are superficial but deep ulcers can happen and the inflammatory polyp is well-explained. The ileocecal valve is normally dilated, and the severity of the ileal inflammation is associated with the severity of cecal inflammation. The characteristic findings are villous atrophy, crypt distortion, mild to moderate chronic active inflammation in the lamina propria with neutrophilic cryptitis, and crypt abscess. In the late stage of backwash ileitis, complete villous atrophy with epithelial regeneration gives rise to a flattened mucosa. Skipped lesions, deep ulcers, fissures, or pseudopyloric metaplasia are not features of backwash ileitis (27-30). 

Celiac Disease

Celiac disease (CD) may look normal on endoscopy and biopsy should be taken from duodenum in suspected cases (31).

The disease can involve distal part of the small intestine and induces intraepithelial lymphocytosis in the ileum. Morphological changes may remain for a long time after following a gluten-free diet (GFD), because serological and endoscopic recovery is faster than histological improvement in the adults with celiac disease who undergo a gluten-free diet (32).

Behcet's Disease 

Behçet's disease demonstrates single or multiple large, round to oval, well-defined edge and fairly flat bottom punched ulcers. The ulcers are mostly situated in the distal ileum and cecum within 50 cm away from the ileocecal valve on the antimesenteric side. The intervening mucosa is usually unremarkable. Necrosis can be induced and form ulcers. The mucosa mostly reveals crypt architectural distortion that may imitate an idiopathic inflammatory bowel disease.

*Mycobacterium avium*

Mycobacterium avium intracellulare (MAI) is spread by inhalation and ingestion into the respiratory and gastrointestinal tract, respectively. An intestinal infection presents with symptoms such as fever, weight loss, abdominal cramps and diarrhea. Endoscopy normally does not show any abnormality. Histologically, the villi could be abnormally blunt or normal, extensively infiltrated with macrophages containing fine, monotonous rods that are PAS, GMS, and acid fast positive. The main differential diagnostic issue is Whipple's disease (25, 29).

Whipple's Disease (WD)

WD is an uncommon bacterial infectious disease of the ileum caused by bacteria Tropheryma Whipplei (Whipple bacillus). Gastrointestinal presentations include weight loss, diarrhea, and lymphadenopathy. Endoscopically, the intestinal mucosa might look normal or may display erosions and yellowish-white patches. The histopathology shows extensive infiltration of the lamina propria by the pink foamy macrophages with intense positive PAS staining along with lamina propria fat collection and epithelial vacuolization. The principal differential diagnoses include MAI infection (Ziehl-Neelsen positive) and muciphages that are faintly PAS (+) with no bacterial inclusions (33-35).

In addition to the aforementioned patterns, there are other pathologic features that can be helpful in the diagnosis (Table 8).

**Table 8:** Other histologic patterns of ileum: Etiologic Associations

Lymphoid hyperplasia
**Giardia lamblia** **Immune-deficiencies** **Other etiologies**
Ischemic ileitis Pattern
**NSAID** **Radiation-induced ileitis**
Pauci Inflammatory Pattern
** Infections** ** GVHD** ** Immunodeficiency** ** Other**
Lamina Propria Infiltrate
**Whipple disease** **Melanosis** **Granulomatous disorders**
Pigments and Extras
**Titanium** **Tattoo Pigment** **Pseudomelanosis** **Formalin Pigment**

Nodular Lymphoid Hyperplasia (NLH)

Nodular lymphoid hyperplasia in the small bowel is an uncommon benign lesion manifested by the presence of numerous small nodules on the surface of the intestine. The nodules are present in the lamina propria and the superficial submucosa of the intestine (Lymphoid hyperplasia of the gastrointestinal tract. Study of 26 cases and review of the literature) (36).

The diagnosis is mainly based on the endoscopic and histological findings, which comprises significant hyperplasia of the lymphoid follicles, mitotically active germinal centres with well-formed lymphocytic mantles (Diffuse follicular lymphoid hyperplasia of the small bowel without primary immunoglobulin deficiency) (37).

This condition does not necessitate any intervention, and treatment is mainly relevant to the associated conditions (38). 

Eosinophilic Pattern

**Fig. 5:**
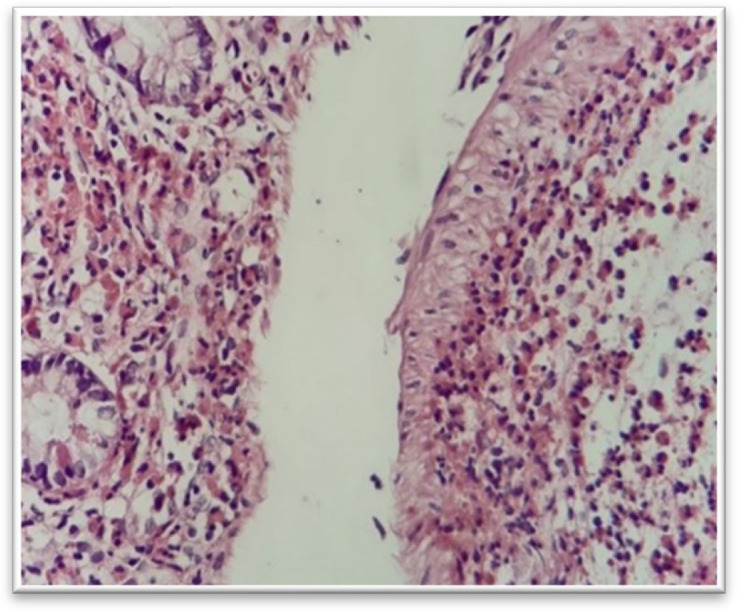
Mucosal eosinophilia, H&E stain, there is a severe eosinophilic infiltration in the subepithelial lamina propria (40 x)

Eosinophils are frequently seen in unremarkable small bowel mucosal sampling. They are scattered in the lamina propria and less commonly in the epithelium. The distinction between the upper limit of normal and abnormally increased tissue eosinophils is not well-determined, but some suggest 5-6/HPF (39-42) (Figure 5).

Eosinophilia should be considered abnormal if the eosinophils form aggregates, permeate the epithelium in more than occasional numbers, or show remarkable degranulation. 

The differential diagnosis of mucosal eosinophilia includes mixed inflammation and inflammatory infiltrates composed mostly or solely of eosinophils (hypersensitivity reactions, infections including parasitic and fungal, eosinophilic gastroenteritis, and Churg-Strauss syndrome).

Eosinophils could be present in any persistent inflammatory condition and in chronic diseases with fluctuation in the severity (42).

The etiological associations of the eosinophilic ileitis pattern can be categorized into the primary and secondary (Table 9).

Ischemic Ileitis Pattern

Embolism, non-occlusive ischemia, arterial thrombosis, and mesenteric vein thrombosis are the most frequent etiologies of the mesenteric insufficiency (43-45).

Arterial insufficiency is the most prevalent cause of the intestinal ischemia. Ischemia can be acute or chronic.

Some of the morphologic patterns of the acute ischemia include superficial epithelial degeneration, necrosis and sloughing, loss of epithelium in the superficial parts of the glands, dilation and congestion of mucosal capillaries, the lamina propria hemorrhage, and hyalinization of the lamina propria owing to the leakage of plasma proteins from the damaged capillaries.

Ischemic changes are formed primarily at the tips of the intestinal villi. With more severe ischemia, mucosal necrosis expands through the whole length of the villi and affects the bases of the crypts. With more severe damage, ischemia involves full thickness of the intestinal wall and may cause bowel perforation.

Chronic ischemic damage displays fibrosis, which can involve all layers of the small bowel and might be accompanied by the hemosiderin deposits and linear ulcers.

**Table 9:** Eosinophilic ileitis pattern: Etiologic Association

Primary (Idiopathic) Eosinophilic Enteritis
**Atopic** **Non-atopic** **Familial**
Secondary Eosinophilic Enteritis
**Medications** **Allergy** **Parasitic Infection** **Inflammatory Bowel Disease** **Connective Tissue Disorders and Vasculitis**

**Pauci Inflammatory Pattern**

**Fig. 6:**
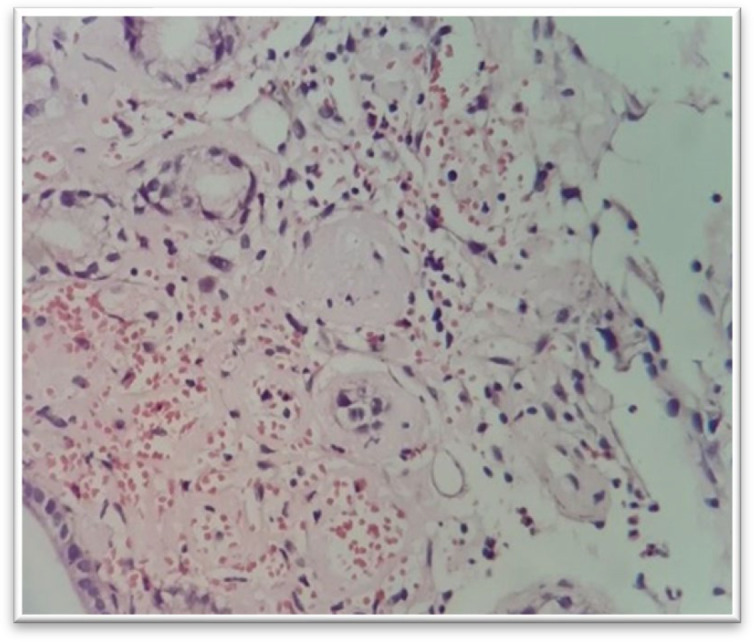
Amyloidosis, H&E stain, biopsy shows amorphous eosinophilic material present around small blood vessels of the mucosa. In the Congo red stain, the material demonstrates an apple-green birefringence (40 x)

Some of the most common causes of the pauci inflammatory injury pattern in the small intestine include infection, drug reaction, graft-versus-host disease (GVHD), scleroderma, amyloidosis, and immunodeficiency (Figure 6).

The important morphologic features that could be helpful in the diagnosis of the mentioned etiologies include viral cytopathic effects, increased apoptosis, mitotic arrest, submucosal fibrosis, and perivascular amyloid deposition.

GVHD is a disorder that is most frequently manifested with this pattern of mucosal injury. Apoptosis and necrosis in the absence of inflammation are characteristic findings of GVHD.

The most commonly involved location in the gastrointestinal tract is the small intestine, followed by the esophagus, stomach, colon or liver (46).

**Lamina Propria Infiltrate**

The most important lamina propria infiltrate in the small bowel include granulomas and diffuse histiocytic infiltrate.

Common causes of granulomatous inflammation of the small bowel are Crohn's disease, tuberculosis, and sarcoidosis.

In the presence of an extensive histiocytic infiltrate in the lamina propria, work up for the atypical mycobacterial infection or Whipple disease should be considered. Acid-fast staining and periodic acid–Schiff staining with diastase digestion (d-PAS) could help find the etiology of the diseases.

**Table 10:** Key Points to remember in ileum disease histopathology

**Goblet cells depletion in the ileum specimen, suggests acute epithelial injury No> 2 crypts normally show crypt branching in a 2mm x2 mm biopsy fragment.**
**Sheets of lymphoid cells in the mucosa or many follicles along the 2 mm length of the muscularis mucosae, are suggestive of a chronic inflammatory bowel disease (IBD) **
**Granulomas are divided regarding the size to the macrogranuloma (>200 μm) and microgranulomas (<100 μm).**
**Mucosal pericryptal microgranulomas mostly are ill-formed and seen in Crohn's disease. The pericryptal mucin granulomas seen in rupture of inflamed crypts should be regarded as differential.**
**Intestinal tuberculosis is more commonly characterized by the presence of multiple confluent non-necrotizing granulomas.**
**Caseous necrosis is rare in tuberculous ileitis. Important causes of necrosis in an ileal mucosal biopsy include invasive fungal infection (mucormycosis), vasculitis, and malignancies.**
**Crypt architectural distortion is an important finding in chronic ileitis.**
Severity of activity should be graded as
**grade 1: activity involving less than 50% of the crypts** **grade 2: activity involving more than 50% of the crypts and ** **grade 3: presence of erosions or ulcers.**
**Eosinophilic infiltration in the mucosa in significant numbers (more than upper limit of 20 ** **eosinophils/HPF) associated crypt destruction or remarkable submucosal or muscularis propria eosinophilic infiltrate raises suspicion of an ** **eosinophilic ileitis.**

Xanthoma is a white/yellowish nodule or plaque on endoscopy, normally asymptomatic and measures 1-10 mm in diameter. It could be present in large numbers and confluent. Microscopic features show collections of foamy, lipid-containing histiocytes within the mildly inflamed lamina propria. No nuclear abnormality and mitoses is present. They are loaded with cholesterol, neutral fats, low-density lipoproteins (LDL) and oxidized LDL. They are periodic acid–Schiff (PAS) and epithelial markers-negative but positive for the CD68. Possible differential diagnoses are poorly cohesive adenocarcinoma, *Mycobacterium avium* intracellulare (MAI), Whipple's disease (WD), and metastatic clear cell renal cell carcinoma.

Small intestinal xanthomatosis can lead to the intestinal obstruction requiring surgical intervention due to a massive tumoral lesion or intestinal motility dysfunction (47). 

**Pigments**

Different endogenous, exogenous, or ingested materials may cause pigment or pigment-like materials to be observed in the intestinal samples.

The most common materials include titanium, pseudomelanosis, tattoo ink, yttrium-labeled micro-spheres, formalin, and melanin pigment.

Titanium is a unique and specific fine dark brown intracytoplasmic pigment deposit of the terminal ileum limited to the cytoplasm of macrophages, also known as the Peyer's patch pigment. Titanium is a food additive used as a whitening agent and accumulates in the terminal ileal lymphoid aggregates of the Peyer's patches.

This pigment may appear in different sizes and shapes pushing the lamina propria constituents aside. Titanium pigment fine granular pattern is a bit different in comparison with the coarse texture of melanin or tattoo pigment (9).

Tattoo ink is mainly used for the endoscopic monitoring, surgical site localization and improving the local lymph node excision. Indian ink is the most frequently used agent so far. It could be identified in the cytoplasm of macrophages mostly in the form of coarse and clumpy granules in comparison with the fine granular texture of titanium.

The preoperative gastrointestinal tattooing is mainly used for localizing the lesions and resulted in improved lymph node excision. Although different types of dyes have been tried, Indian ink is the most applied agent so far (48, 49).

Pseudomelanosis is a misnomer, and the pigment is mainly iron instead of melanin and mixed with a variable amount of other chemical elements, including calcium.

Pseudomelanin is a coarse, dark-brown pigment that is highlighted by iron or calcium stains. It is caused by the gastrointestinal bleeding, special drugs like iron pills or anti-hypertensive agents. Renal failure history may exist.

It is a uniform granular brownish cytoplasmic pigment located at the villi tips and is only visible endoscopically in 36% of the cases (50-54).

Formalin represents as worrisome lamina propria deposits but lacks clinicopathological importance and is appreciated as a dark brown, granular, birefringent, and refractile artifact.

Unlike the aforementioned pigments, it is not limited to the cytoplasm of macrophages. It can be found at both intra- and extra-cellular locations.

Bizarre shapes and forms are a characteristic histological feature of the formalin deposits. Under light microscopy, depositions are not on the same plane, which could be applied as a helpful reliable distinguishing finding (55-57).

Among other pigments that we may encounter, melanin should not be missed or misdiagnosed.

The aforementioned pigments are appreciated in macrophages with rather nearly normal nuclei, but this one is mostly found in neoplastic cells having abnormal hyperchromatic pleomorphic nuclei, prominent nucleoli, and surrounding atypical mitotic figures or necrosis.

Characteristically, it represents a coarsely granular brownish-black pigment that can be highlighted by the special stains like Fontana-Masson or melanocytic markers reactivity like S100 or Melan-A.

Yttrium-labeled micro-spheres are a widely used targeted therapy for the surgically unresectable malignancies, but non-target tissues and organs may be affected as a result of inadvertent and unintended use.

The associated background radiation injury may be appreciated, i.e., stromal hyalinization, bizarre cells and dilated vascular channels.

Uniform dark purple, round, spherical radio emission agent ranging from 30 to 40 μm for up to 2 weeks post administration is the histologic findings (14).

In all cases, immunohistochemistry (IHC) staining must be considered for the Cytomegalovirus (CMV) detection for two reasons: first, radiation atypia may obscure viral cytopathy, and second, almost all patients must be considered immune deficient. 

Regarding the wide spectrum of the diseases occurring in the ileum, reviewing a summary of the key morphological changes is always helpful (Table 10).

## Conclusion

Nowadays, terminal ileoscopy (TI) is a necessary part of the colonoscopy. It confirms fulfilment of colonoscopy. TI adds only three minutes to the duration of colonoscopy procedure. Moreover, there are no additional complications to those of colonoscopy.

Pathologists around the world are facing an increasing number of ileal biopsy specimens. A good knowledge about the indications, clinical features, and microscopic findings as well as the interpretation of the light microscopic and ancillary laboratory tests can help us to narrow down the possible differential diagnoses. 

## Ethics approval & Consent to Participate

Not Applicable. 

## Authors' contributions

AM: Conception of the work and critical revision of the article. AMP: Searching, Data collection and Article edition. AY: Searching, Data Collection. KK: Searching, Data Collection. LBK: Searching, Data Collection, Drafting the article, Critical revision of the article and final approval of the version to be published.

## Conflict of Interest

The authors declared no conflict of interest.

## Funding

The author(s) received no financial support for the research, authorship, and/or publication of this article.
